# Charlatanry in Destructive Diseases

**Published:** 1877-10

**Authors:** 


Charlatanry in Destructive Diseases.—When the religious press opens its columns to the advertisements and paid laudators of practioners who employ secret remedies, it seems to me to be participating in one of the grossest frauds of modern civilization. I say religious press; for secular papers, being regarded as newspapers, are not so fully credited, are not so influential, especially with the religious public, as the religious presses are. The secular press is not held to such strict accountability as the former. But even for the former there is a class of advertisements forbidden by law, because they give publicity to infamous employments. A lottery even can not be advertised without a penalty, because of its fraudulent workings.

The publishers of the religious press exculpate themselves from participating in the wrong of advertising the cures of charlatans, by saying that they are not responsible for the truth or value of the thing noticed. If this be sound doctrine, the theater, circus, and every other amusement, may be given currency in a religious journal. Indeed, the religious press can get double price for this class of advertisements, for their pious readers behold it as a *quasi* indorsement; and it is because of this implied recommendation that cancer and consumption doctors patronize religious papers so assiduously.

What is the nature of an advertisement of a secret remedy for cancer and consumption? Let us see. A cancer is the most terrible of all diseases to which the human form is liable. It pervades the whole earth, attacking the poor, the rich, both sexes alike. Its development in any one is a horror. Its *cure*, therefore, by any remedy or system of practice, would be a boon of incalculable value. To discover a remedy and promulgate it, would be one of the grandest performances of man. Such an achievement would cover the whole life of such a man with the benediction of suffering humanity—load him with rewards, and "gild his sepulcher and embalm his name." It would answer, in short, all the powerful instincts we possess of pity, mercy, honor, wealth and fame. Like Jenner's renown, it would grow with the age.

From the knowledge we possess of human instincts and practice to relieve suffering and even danger, periling, as men do, health and life in such divine acts, we have no hesitation in asserting that no human being, acquiring either by laborious research, or by purchase, or in any way, a method of curing cancer or any destructive disease, *could keep so fearful a secret.* He must publish it to the whole race as an act of mercy and love; his human heart would not permit him to do otherwise. There is but one excuse for the man who could conceal so important a secret, and that is that he is a *moral idiot!* No, the *claim* of so invaluable a secret remedy is a positive proof that it is a gross fraud, for only a fiend could keep it.

But the cancer doctor has an excuse, and it is this: "Am I not entitled to the reward of my discovery and skill, like any other inventor or creator of useful knowledge? If I tell my method and teach it broadcast, it would give every one the same opportunity, and people would not come to me from a distance—come to *me* as the great cancer (or consumption) doctor."

The creator of a book has a copyright, but the book is sold everywhere, and any one can read it. The inventor of a machine has a patent, and gets a royalty. There is no secret, however, in these cases; all can get the benefit by purchase at their own doors: it is not necessary for the people who have need of these things to go at great expense to a distant place to obtain them. Now, I will admit that a remedy disclosed, and the practice with it, can not be treated exactly as the question of the book or the machine; but this I can say, that if any man has a remedy, and knows its application cures cancer, if he will go to the capitals of nations—christian or pagan—he can procure for the disclosure and demonstration millions of money, and honors and titles of distinction besides. No, it is impossible to urge the money question as a reason for keeping the dread secret. How, then, can a man demand what it is impossible to accomplish, namely, that people from remote places must come to him for treatment? I repeat, the pretense of a secret cure is a tacit confession of fraud in a moral sense.

From all who think that they have received any benefit by the prescriptions of these charlatans, certificates are obtained, which are published; generally they are untruthful, unless the disease for which they had been treated was simple in its character. But how many, who have been cheated and robbed, go away in silence and despair, and nothing is heard of their disappointment.

I have now sufficiently shown, I trust, that the self-assumed possessors of cures of cancer and consumption violate the common instincts of humanity, whether represented by love of money, pity, benevolence, honor or fame; all of which are immeasurably more gratified by publicity of methods of treatment than by their concealment. And I therefore re-assert, without a fear of successful contradiction, that all those who assume, or participate in the assumption, that a secret cure for cancer or consumption exists, are parties to the basest fraud of modern times.

Finally, when it is announced that such charlatan has been a regular physician, and is a graduate of a regular medical school, it is an attempt to cover his nefarious practices with the indorsement of a humane and self-sacrificing profession—one that abhors such secrets—one that perils itself in the face of pestilence and war to aid suffering; and every honest school, when its title to honor has become degraded by one who announces the possession of a secret remedy for the cure of a dire disease, and refuses to disclose it, hastens to erase his name from its honorable enrollment, and his profession spews him out of its mouth.—*From American Practitioner.*

